# Population Difference in the Associations of *KLOTH* Promoter Methylation with Mild Cognitive Impairment in Xinjiang Uygur and Han Populations

**DOI:** 10.1371/journal.pone.0132156

**Published:** 2015-07-21

**Authors:** Mei Luo, Xiaohui Zhou, Huihui Ji, Wenjuan Ma, Guili Liu, Dongjun Dai, Jingyun Li, Lan Chang, Lei Xu, Liting Jiang, Shiwei Duan, Qinwen Wang

**Affiliations:** 1 Department of Internal Medicine for Cadres, the First Affiliated Hospital of Xinjiang Medical University, Urumchi, 830000, China; 2 Ningbo Key Lab of Behavior Neuroscience, Zhejiang Provincial Key Laboratory of Pathophysiology, School of Medicine, Ningbo University, Ningbo, Zhejiang, 315211, China; Nanjing University of Aeronautic and Astronautics, CHINA

## Abstract

**Background:**

Mild cognitive impairment (MCI) is the intermediate stage of the cognitive changes between normal aging and dementia. *KLOTH* is an age-related gene that may contribute to the risk of MCI. The aim of our study was to explore the association between *KLOTHO* promoter methylation and MCI in Xinjiang Uygur and Han populations.

**Methods:**

DNA methylation assay was performed using the bisulphite pyrosequencing technology among 96 Uygur (48 MCI and 48 controls) and 96 Han (48 MCI and 48 controls) Chinese individuals from Xinjiang province of China.

**Results:**

We found significant association between *KLOTHO* promoter methylation and MCI in the Han Chinese (CpG1: p = 3.77E-06; CpG2: p = 1.91E-07; CpG3: p = 5.83E-07; CpG4: p = 2.23E-05; CpG5: p = 3.03E-06) but not in the Uygur Chinese. Higher *KLOTHO* promoter methylation levels were found in Han MCI patients than Uygur MCI patients for all the five CpGs (adjusted p values by age < 0.02).

**Conclusion:**

Our results showed that *KLOTHO* promoter hypermethylation contributed to the MCI risk in Xinjiang Han Chinese but not in Xinjiang Uygur Chinese. The population difference of *KLOTHO* methylation in the risk of MCI required further investigation in the future.

## Introduction

Mild cognitive impairment (MCI) refers to the intermediate stage of the cognitive changes between normal aging and dementia [1]. Individuals with MCI showed cognitive impairment greater than expected for their age and education, but their mental functions are relative complete and do not reach the criteria for dementia. About 10–15% of MCI persons develop into dementia (mostly Alzheimer’s disease, AD) per year, in constrast of 1–2% AD incidence in the general population per year [2]. The incidence of MCI ranges from 1% to 6% per year, while the prevalence ranges from 3% to 22% per year [3, 4] in the Western countries. In China, the prevalences of MCI in different regions vary between 5.4% (Taiyuan city) and 25% (Shaanxi province) [5]. Nowadays, MCI has become a major threat to the eldly, and it should be early detected and intervented in order to delay or prevent the occurrence of dementia.

*KLOTHO* is a longevity and neuroprotective gene [6], encoding a single-pass transmembrane protein with a long extracellular domain and a short cytoplasmic tail [7]. KLOTHO controls multiple growth factor signaling pathways, including insulin, IGF-1 and Wnt [8]. Klotho is able to protect cells and tissues from oxidative stress by stimulating the expression of antioxidant proteins [9]. Klotho-mutated mice had cognition impairment due to an increased oxidative damage to hippocampus neurons [10], and the similar result was also observed in rhesus monkey [11, 12]. Lower KLOTHO concentration was discovered in the cerebrospinal fluid of older adults, especially in the AD patients [13]. Our previous study indicated lower KLOTHO protein levels in MCI patients than controls [14].

Alteration in promoter methylation is often found to affect gene expression [15] and was shown to be associated with AD [16], and other diseases [17–20]. MCI is a complex disease affected by both genetic and environmental factors [21]. Epigenetics is considered as a link between genetics and environment, and DNA methylation as a major part of epigenetics may play an important role in the development of MCI [22]. Aberrant expression of some important genes, such as APOE [23], presenilin 1 and APP [24], were shown to be associated with the occurrence of MCI. However, the relationship between *KLOTHO* methylation and MCI is still unclear. Therefore, we examined whether *KLOTHO* promoter methylation was associated with MCI in the Han and Uygur Chinese in Xinjiang province of China.

## Materials and Methods

### Samples and clinical data

All the participants over 60 were selected in this study. Among them, there were 96 MCI patients (48 Han and 48 Uygur) and 96 well-matched controls (48 Han and 48 Uygur) collected from epidemiological surveys between 2010 and 2014 in Xinjiang province of China. According to the Diagnostic and Statistical Manual of Mental Disorders 4th edition, the inclusion criteria for MCI comprised the following items: 1) memory complaint; 2) GDS scores were 2–3 grade and MMSE scores for illiterate, primary school and secondary school (and above) were 18–21, 21–24, and 25–27, respectively; 3) decreased daily activities and social participation; 4) HIS score ≤ 4 and no specific causes of cognitive decline; 5) cognitive impairment lasted for over three months; 6) absence of dementia. Subjects were excluded if they had history of mental illness or mental retardation or suffering from severe heart or lung or kidney dysfunction, severe endocrine disease, severe infectious diseases, and toxic encephalopathy. Subjects were also excluded if they had brain dysfunctions in the past 6 months, including stroke, Parkinson's disease, brain tumors, depression, a history of head trauma, or a history of psychotropic drug use, alcohol, or drug addiction. All the collected individuals were Han or Uygur Chinese from Xinjiang in the Western China. All the subjects voluntarily participated in this study. Written informed consent was obtained from each of the subjects following a complete description of the study. The institutional ethics committee of the First Affiliated Hospital at Xinjiang Medical University approved this study.

### Biochemical analyses

Nucleic acid was extracted from the blood samples using the Genomic DNA Mini Kit (TIANGEN; Beijing, China). The DNA concentration was measured by the ultramicro nucleic acid ultraviolet tester (NANODROP 1000, Wilmington, USA). Plasma levels of biochemical factors (including triglyceride (TG), total cholesterol (TC), high density lipoprotein (HDL), low density lipoprotein (LDL)) were detected by biochemical laboratory (Beckman; Brea, CA, USA). DNA methylation levels of each CpG sites were tested by pyrosequencing technology. Sodium bisulphite (EZ DNA Methylation-Gold Kit; ZYMO RESEARCH, Orange County, California, USA) was used to convert all unmethylated cytosines to uracils, and meanwhile the methylated cytosines unchanged. Polymerase chain reaction (PCR) (Zymo Taq PreMix, ZYMO RESEARCH, Orange County, California, USA) was used to augment target DNA sequences. The primers were all designed by PyroMark Assay Design software. The sequences were 5’-TTGGGTTTTAGAGTGGGAGAAAAGT-3’ (forward primer), 5’-Biotin-AAACCCTCAAATTCATTCTCTTTACCTACC-3’ (reverse primer), and was 5’-AGTGAGAGTAGGTGT-3’ (sequencing primer). All the oligomers were synthesized by the Sangon Biotechnology company (Shanghai, China).

Statistical Program for Social Sciences (SPSS) software 16.0 (SPSS, Inc., Chicago, IL, USA) was used in this study and a *p* value < 0.05 was considered to be significant. Nonparametric testing was used to compare differences in the each CpG sites of continuous variables between the MCI cases and controls. Spearman rank correlation test was used to analyze the associations between *KLOTH* methylation and metabolic characteristics of MCI subjects.

## Results

**Fig 1:**
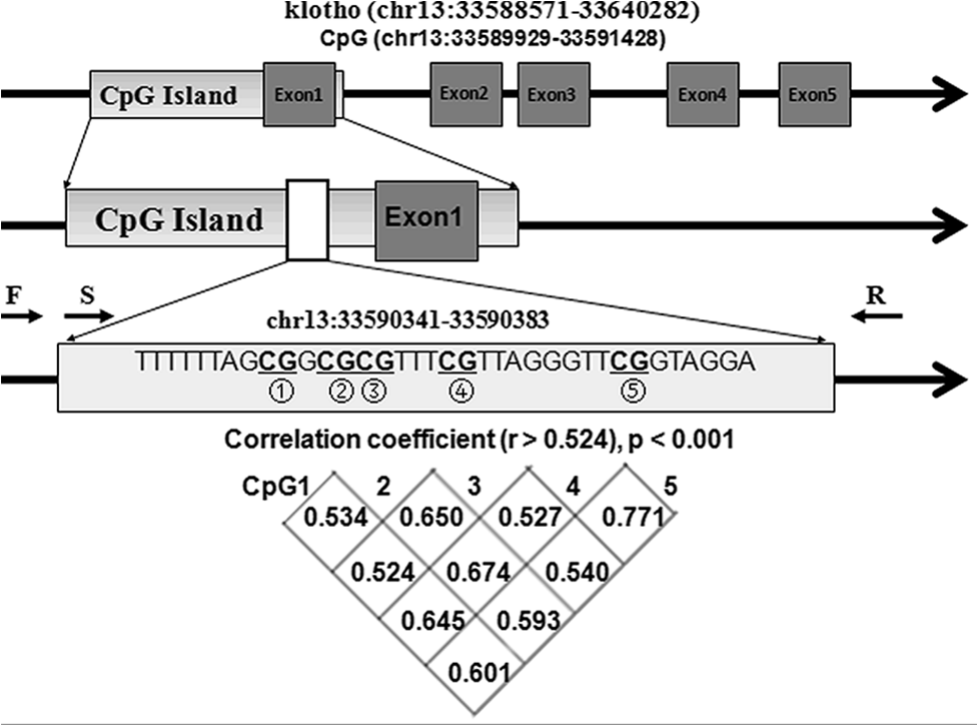
The locations of the five *KLOTH* promoter CpG sites. Note: F stand for Forward primer, R stand for Reverse primer, S stand for Sequencing primer.

**Fig 2:**
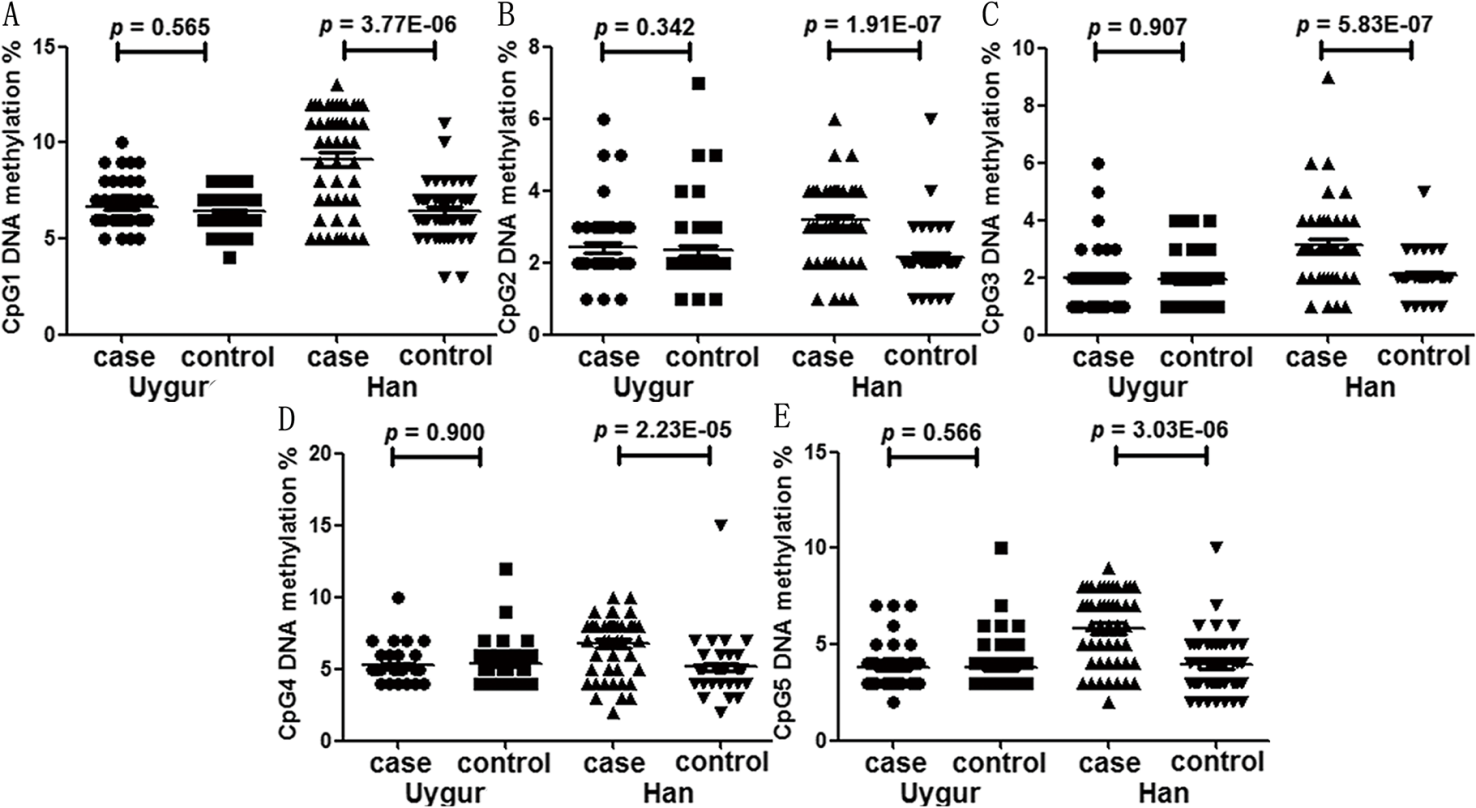
Association between *KLOTHO* methylation and MCI.

A total of five CpGs on the *KLOTHO* promoter were assessed to explore their associations with the risk of MCI (Fig 1). Significant correlation was found among the methylation levels of the five CpGs (r > 0.524, p < 0.001). As shown in Table 1 and Fig 2, significantly elevated *KLOTHO* promoter methylation was found in MCI cases than controls in Xinjiang Han Chinese population (CpG1: *p* = 3.77E-06; CpG2: p = 1.91E-07; CpG3: p = 5.83E-07; CpG4: p = 2.23E-05; CpG5: p = 3.03E-06) but not in Uygur Chinese population. Similar results in the female and male Xinjiang Han Chinese (male: CpG1: *p* = 0.001; CpG2: *p* = 2.36E-04; CpG3: *p* = 0.003; CpG4: *p* = 0.007; CpG5: *p* = 0.002, female: CpG1: *p* = 0.001; CpG2: p = 3.23E-04; CpG3: p = 1.89E-05; CpG4: p = 0.001; CpG5: p = 4.184E-04, Table 2).

**Table 1:** Characteristics of *KLOTHO* methylation and important parameters in the Xinjiang Uygur and Han Chinese.

	Uygur			Han		
	Case(n = 48)	Control(n = 48)	p value	Case(n = 48)	Control(n = 48)	p value
BMI	23.59±3.76	23.12±4.14	0.57	24.49±2.78	24.49±3.33	0.986
Age(years)	70.83±4.54	71.02±4.42	0.838	77.41±5.52	77.70±5.53	0.797
CpG1	6(6,7)	6(6,7)	0.565	10 (7,11.75)	6 (6,7)	**3.77E-06**
CpG2	2(2,3)	2 (2,2)	0.342	3(3.25,4)	2 (2,2)	**1.91E-07**
CpG3	2(1,2)	2(1,2)	0.907	3(2,4)	2 (2,2)	**5.83E-07**
CpG4	5(5,5)	5(5,6)	0.900	8(5,8)	5 (5,5)	**2.23E-05**
CpG5	4(3,4)	3(3,4)	0.566	6(4,8)	4 (3,5)	**3.03E-06**

**Table 2:** Subgroup characteristics of *KLOTHO* methylation and important parameters in the Xinjiang Uygur and Han Chinese.

	Uygur			Han		
	Case(n = 24)	Control(n = 24)	p value	Case(n = 24)	Control(n = 24)	p value
male						
BMI	24.96±4.32	23.72±3.53	0.283	25.34±2.82	24.15±2.37	0.121
Age(years)	72.00±5.00	72.46±4.46	0.739	78.33±6.32	78.71±6.27	0.837
CpG1	6(6,7)	7(6,7)	0.469	10(7,11.75)	6(6,7)	**0.001**
CpG2	2(2,3)	2(2,2)	0.928	3(3,4)	2(2,2)	**2.36E-04**
CpG3	2(1,2)	2(2,2)	0.217	3(3,4)	2(2,3)	**0.003**
CpG4	5(5,5)	5(5,6)	0.484	7.5(5,8)	5(4,5)	**0.007**
CpG5	3(3,4)	3(3,4)	0.572	6(4,7)	4(3,5)	**0.002**
female						
BMI	22.21±2.53	22.53±4.69	0.774	23.64±2.52	24.85±4.11	0.226
Age(years)	69.67±3.81	69.58±3.98	0.941	76.50±4.55	76.71±4.60	0.875
CpG1	6.5(6,7)	6(6,7)	0.125	9.5(6,11.75)	6(5.25,7)	**0.001**
CpG2	2(2,3)	2(2,2)	0.228	3(2,4)	2(2,2)	**3.23E-04**
CpG3	2(2,2)	2(1,2)	0.123	3(2,4)	2(2,2)	**1.89E-05**
CpG4	5(5,5)	5(4,5)	0.518	8(5,8)	5(5,5)	**0.001**
CpG5	4(3,4)	3(3,4)	0.149	7(4,8)	4(3,4)	**4.184E-04**

**Fig 3:**
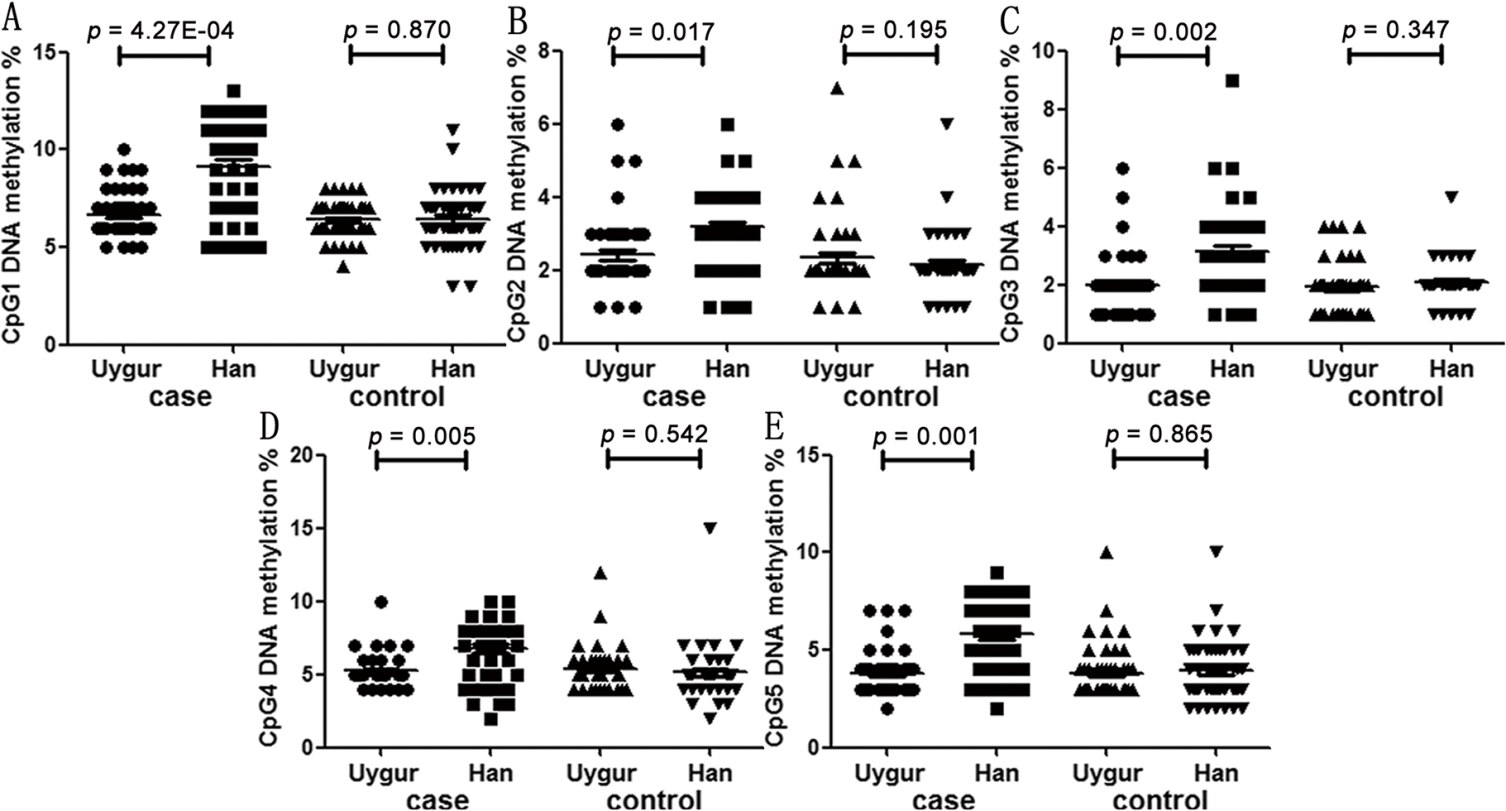
Population difference of *KLOTHO* methylation.

Since there were significant differences in the age between Uygur and Han samples (MCI cases: p = 6.78E-09; controls: p = 3.22E-09; Table 3), thus the population differences of various parameters were adjusted by age. According to the Table 3 and Fig 3, Han MCI patients had significantly higher levels of *KLOTHO* promoter DNA methylation than Uygur MCI patients (CpG1: adjusted p = 4.27E-04; CpG2: adjusted p = 0.017; CpG3: adjusted p = 0.002; CpG4: adjusted p = 0.005; CpG5: adjusted p = 0.001).

**Table 3:** Tests for population differences of *KLOTHO* methylation and important parameters.

	case				control			
	Han	Uygur	p value	adjusted p value (by age)	Han	Uygur	p value	adjusted p value (by age)
BMI	24.49±2.78	23.58±3.76	0.186		24.49±3.34	23.12±4.14	0.077	
Age(years)	77.42±5.52	70.83±4.54	**6.78E-09**		77.71±5.53	71.02±4.42	**3.22E-09**	
CpG1	10(7,11.75)	6(6,7)	**1.39E-05**	**4.27E-04**	6(6,7)	6(6,7)	0.973	0.870
CpG2	3(2.25,4)	2(2,3)	**1.03E-04**	**0.017**	2(2,2)	2(2,2)	0.288	0.195
CpG3	3(2,4)	2(1,2)	**1.11E-06**	**0.002**	2(2,2)	2(1,2)	0.126	0.347
CpG4	8(5,8)	5(5,5)	**7.97E-05**	**0.005**	5(5,5)	5(5,6)	0.333	0.542
CpG5	6(4,8)	4(3,4)	**9.04E-07**	**0.001**	4(3,5)	3(3,4)	0.668	0.865

**Fig 4:**
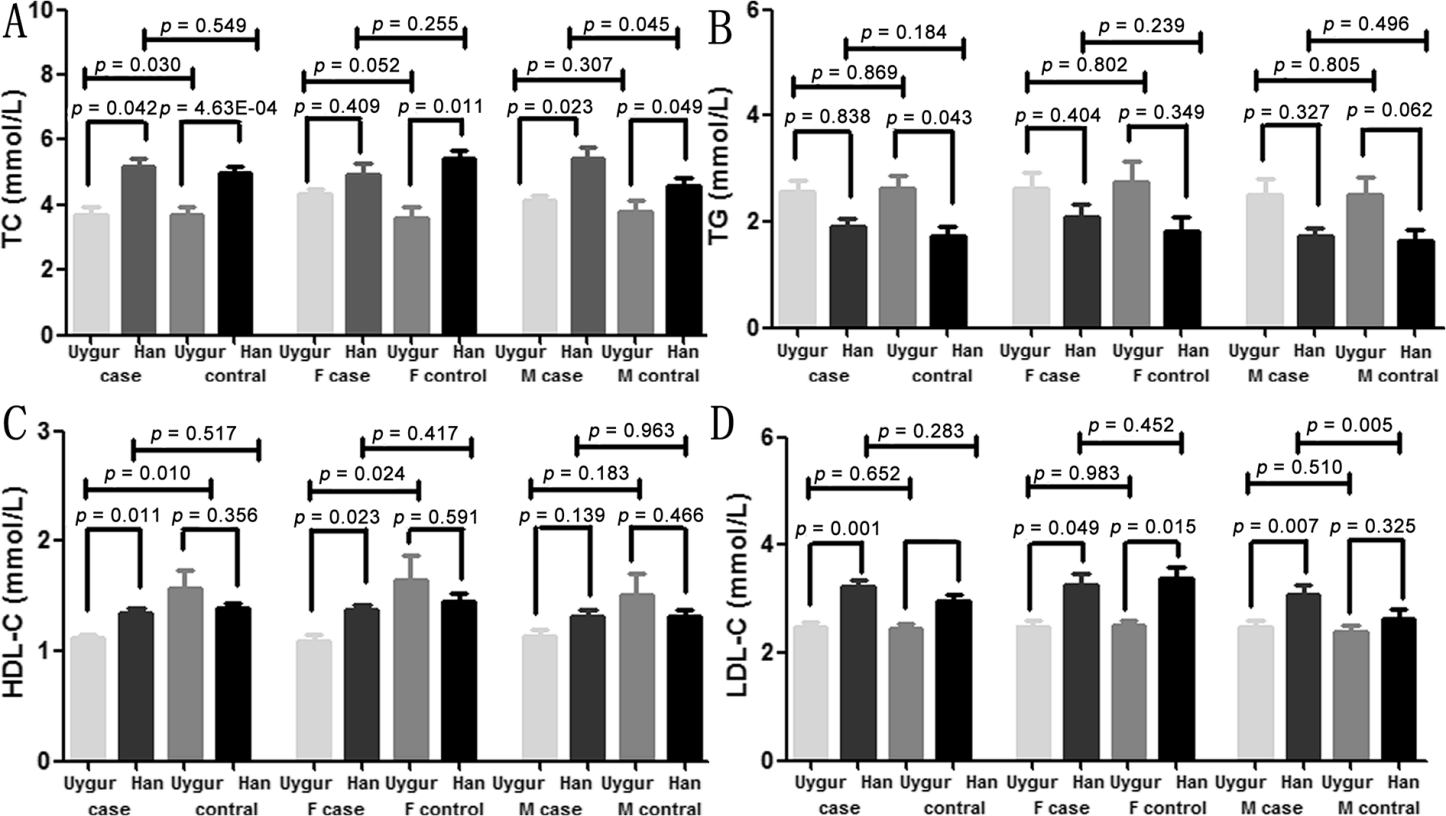
Differences of important parameters between Xinjiang Uygur and Han populations.

Among the 5 phenotypes, significantly higher TC and lower HDL-C levels were found in the Uygur MCI patients than the Uygur controls (TC: p = 0.030; HDL-C: p = 0.010, Fig 4). Lower levels of HDL-C were found in the Uygur MCI females than the Uygur female controls (p = 0.024, Fig 4). In the Xinjiang Han Chinese, significantly higher levels of TC and LDL-C were found in the male MCI patients compared to the male controls (TC: p = 0.045, LDL-C: p = 0.005, Fig 4).

After adjusted by age, significantly higher levels of TC, LDL-C, and HDL-C were found in Han MCI patients than Uygur MCI patients (TC: adjusted p = 0.042; LDL-C: adjusted p = 0.011; HDL-C: adjusted p = 0.011, Fig 4). Significantly lower TG and higher TC and LDL-C levels were found in Han controls than Uygur controls (TG: adjusted p = 0.043; TC: adjusted p = 4.63E-04; LDL-C: adjusted p = 0.008, Fig 4). Besides, significantly higher levels of LDL-C were found in Han patients than Uygur patients (adjusted p = 0.049, Fig 4).

As shown in the Table 4, statistically higher levels of CpG sites were found in Han MCI patients than Uygur MCI patients in the male and female subgroups (male: CpG1: adjusted p = 0.009; CpG2: adjusted p = 0.047; CpG3: adjusted p = 0.009; CpG5: adjusted p = 0.034, Table 4; female: CpG1: adjusted p = 0.012; CpG4: adjusted p = 0.017; CpG5: adjusted p = 0.006, Table 4). In the male subgroup, the levels of TC and LDL-C were all significantly higher in Han patients compared to Uygur patients (TC: adjusted p = 0.023; LDL-C: adjusted p = 0.007, Fig 4). Significantly higher TC levels were also found in the male Han controls compared to the male Uygur controls (adjusted p = 0.049, Fig 4). In the females, significantly higher levels of HDL-C were found in the Han MCI patients than the Uygur MCI patients (adjusted p = 0.023, Fig 4). Statistically higher levels of TC and LDL-C were found in Han female MCI compared to Uygur female controls (TC: adjusted p = 0.011; LDL-C: adjusted p = 0.015, Fig 4).

**Table 4:** Tests for gender differences of *KLOTHO* methylation and important parameters.

	case				control			
	Han	Uygur	p value	adjusted p value (by age)	Han	Uygur	p value	adjusted p value (by age)
male								
BMI	25.34±2.82	24.96±4.32	0.721		24.15±2.37	23.72±3.53	0.625	
Age(years)	78.33±6.31	72.00±4.50	3.60E-04		78.71±6.27	72.46±4.46	2.44E-04	
CpG1	10(7,11.75)	6(6,7)	**4.11E-04**	**0.009**	6(6,7)	7(6,7)	0.897	0.888
CpG2	3(3,4)	2(2,3)	**0.003**	**0.047**	2(2,2)	2(2,2)	0.291	0.533
CpG3	3(3,4)	2(1,2)	**3.73E-04**	**0.009**	2(2,3)	2(2,2)	0.388	0.756
CpG4	7.5(5,8)	5(5,5)	**0.012**	0.121	5(4,5)	5(5,6)	0.215	0.729
CpG5	6(4,7)	3(3,4)	**4.03E-04**	**0.034**	4(3,5)	3(3,4)	0.920	0.936
female								
BMI	23.64±2.52	22.21±2.53	0.057		24.85±4.11	22.53±4.69	0.075	
Age(years)	76.50±4.54	69.67±3.81	**9.88E-07**		76.71±4.60	69.58±3.98	**7.11E-07**	
CpG1	9.5(6,11.75)	6.5(6,7)	**0.010**	**0.012**	6(5.25,7)	6(6,7)	0.871	0.778
CpG2	3(2,4)	2(2,3)	**0.016**	0.215	2(2,2)	2(2,2)	0.692	0.181
CpG3	3(2,4)	2(2,2)	**0.001**	0.105	2(2,2)	2(1,2)	0.128	0.39
CpG4	8(5,8)	5(5,5)	**0.003**	**0.017**	5(5,5)	5(4,5)	0.973	0.897
CpG5	7(4,8)	4(3,4)	**0.001**	**0.006**	4(3,4)	3(3,4)	0.510	0.645

## Discussion

In the current study, we investigated the association between *KLOTHO* promoter methylation and MCI in two Xinjiang populations. In the Xinjiang Han, significant results were found in the overall analysis and the gender-based subgroup analyses, although no positive results existed in the Uygur population. Significantly higher level of *KLOTHO* promoter methylation was found in Han patients compared to Uygur patients. In addition, population differences were also found in multiple clinical phenotypes including the levels of TG, TC, HDL-C and LDL-C. Different dietary cultures in two populations might explain the population differences of the above phenotypes.

*KOLOTHO* is a longevity gene and has been considered to be involved in MCI which is an age-related disease. *KOLOTHO* mainly expressed in brain and kidney, secreting into CSF and serum, respectively [25]. *KLOTHO* as a related factor to oxidative damage has the ability to protect neurons in brain [26]. *KLOTHO* was considered as an aging suppressor gene [27], and over expression of *KLOTHO* significantly extended the life span of mice [28, 29].Meanwhile, we found a higher methylation level in *KLOTHO* promoter in the MCI patients of Xinjiang Han population than matched controls, and the same results were also observed in the subgroup stratified by gender. No difference was found in Uygur population. DNA methylation alteration of promoter was often found to influence gene expression ^[^ ^30^ ^]^. In addition, hypermethylation of *KLOTHO* was shown to low expression in hepatocellular carcinoma tissues [31]. Repressions of *KLOTHO* migth have an adverse effect for MCI patients according to a previous study [27]. Hence, we hypothesized that hypermethylation of *KLOTHO* promoter might play a role in the development of MCI of Xingjian Han population via the pathway of down regulation of *KLOTHO* expression.

Population difference could change gene activity as described previously [32]. Populations are complicated in Xinjiang Province. Uygur population and Han population belong to Caucasian and Mongolian, respectively [33]. Previous study had investigated significant association between IL-4, IFN-gamma promoter methylation and allergic rhinitis patients of Uygur and Han Chinese in Xinjiang [34]. Significantly higher DNA methylation levels of microRNA-375 promoter were found in Han T2D patients than Kazak T2D patients in Xinjiang [35]. In this study, significantly higher level of *KLOTHO* promoter methylation was discovered in Han MCI patients than Uygur patients, suggesting a population specific mode of *KLOTHO* methylation in the susceptibility of MCI.

Dyslipidemia is associated with both the occurrence and progression of MCI. HDL-C and TC were belong to vascular risk factors and could increase the risk of MCI and the risk of conversion from MCI to AD [36, 37]. A correlation between HDL-C and dementia was also revealed in the elderly [38]. A cross-sectional study about cognitive impairment suggested that TC and LDL-C were independent risk factors for MCI [39]. An interdisciplinary longitudinal study showed that higher TC levels were associated with an increased risk for cognitive disorders [40]. Higher TC and lower HDL-C levels were found in Chinese T2D patients with MCI compared to simple T2D patients [41]. Consistent with the previous findings, our study showed a significantly higher TC level in MCI compared to controls in the Uygur population.

Population differences were also found in four phenotypes including TG, TC, HDL-C and LDL-C. Higher levels of TC and LDL-C were found in Han population than Uygur population in both MCI and control subgroup. However, elevation of HDL-C level was only found in the Han MCI patients than Uygur MCI patients. In addition, significantly lower levels of TG were found in Han patients and controls than Uygur patients and controls. These phenotypes are shown to be influenced by many factors including genetic factors, eating habit, and lifestyle [42, 43]. The population differences in both the genetic and the environmental factors might explain the differences of above phenotypes in two populations.

Ethnic difference has been observed in the diagnosis, clinical manifestation, disease management, prevalence, and cause of MCI. Race difference was found in the memory performance even after adjusting by the status of education, depression, gender, and memory complaints [44]. As the measurement of cognitive functioning, Hachinski ischemic scale scores were shown to differ by ethnicity [45]. African American MCI patients were found to have faster rate of cognitive decline compared with non-African American ones [46]. Ethnic disparities were also observed in the management of MCI patients [47]. Caucasian MCI patients underwent significant more neuropsychologic testing; while African-Americans had more depression screening tests and were less likely to be prescribed with acetylcholinesterase inhibitors [47]. The prevalence of dementia has been reported to be higher among African Americans and Caribbean Hispanics, lower among Japanese Americans, and similar among Native Americans and Mexican Americans compared with non-Hispanic whites [48]. English usage in Australia was shown to be related to to lower prevalence of MCI and higher rates of reversion from MCI to normal [49]. In the United States, more African American and Hispanic MCI patients were attributable to diabetes than non-Hispanic white MCI patients [50]. Racial disparities in financial capacity were found to exist among patients with amnestic MCI [51]. Predictive risk factors for MCI in non-Hispanic cohorts were not associated with MCI in Mexican Americans [52], who were more likely to be possessed the ApoE ε4 allele less frequently [52]. In addition, ApoE ε4 allele was protective for MCI only in Han Chinese but not in Hui Chinese [53].

In summary, our study identified the contribution of *KLOTHO* promoter methylation to MCI in Xinjiang Han population. The lack of significant results in the Xinjiang Uygur population might be due to the differences in genetics and environment. Further investigation on the population difference was needed in the future.
